# Dental Jurisprudence

**Published:** 1870-10

**Authors:** 


### Editorial.

### DENTAL JURISPRUDENCE.

In a recent murder trial in this State (Ohio), known as the Lunsford murder case, there were some points of interest to the Dental profession. Upon the arm of the victim when found there were the imprints of teeth, made by biting, which it was presumed were made at the time of the murder, and as was claimed by the prosecution, by the prisoner at the bar. The question whether the biting, judging from its appearance, was done at the time of the murder, before it, or after, was one that elicited some investigation and discussion. It was intimated that the character of the contusions would indicate to the close observer clearly the facts in this respect. However clearly any one may have supposed he saw evidence of this fact, the jury failed to be convinced further than that the biting was done at some time not very remote from the murder. We presume that by a thorough investigation of the wounds and the contiguous parts after death and before decomposition had set in, it would not have been very difficult to determine whether the wounds had been made before death, while all the vessels of the body had the normal amount of blood in them, or after death, when, as in this case, at the finding of the body, the system was drained of its blood. There would undoubtedly upon minute examination be a palpable difference in the character of the wounds.

As to whether the injury was done at the time of the murder, or some time previous, could only be judged of by the indications of more or less struggling and resistance, which would be indicated by the part in the grasp being drawn through the clenched teeth. Some wounds or contused spots on the soft part of the arm, below the elbow, indicated by the extension of the echymosis that it had been drawn from between the teeth while the grasp was upon it.

A seizure was made upon the wrist of the victim from which she could not release herself, and the marks of teeth were accurately defined upon the skin. When the comparison was made of the teeth of the prisoner with these marks, the indentations were all removed by the elasticity of the parts, and only the echymosed spots showed the place of the bite. This comparison was made more than forty-eight hours after the murder.

The prisoner had the four superior incisors and the left superior cuspid tooth standing; and the right superior cuspid, the bicuspids, and some of the molars on each side missing. The standing cuspid was a little shorter than its fellows, the contiguous lateral incisor was perhaps one-third to one-half a line longer than either of the adjoining teeth; the left central was a little shorter than the lateral or central incisor next to it, and the right central incisor was just about the same length as the contiguous lateral incisor. The left central was turned a little, perhaps about fifteen degrees, so that its edge was thus much out of its proper position, or the true circle of the edges of the teeth.

Now it was in evidence that the correspondence between these teeth and the contused points of the last mentioned bite upon the wrist was perfect, this correspondence being very acccurate in several respects; first, then, five echymosed spots; second, they correspond in position with the edges or points of the five teeth, both in respect to the circle and the position of the edges; and third, it was further shown that the deeper contused spots corresponded with the longer teeth, except that of the cuspid tooth, which had caught upon the head of the ulna, and though it was shorter than its fellows, had the opportunity to make a more definite bruise. Now it was claimed that these facts indicated clearly and unmistakably that this wound had been made by the teeth of this prisoner, that the distinctive features and peculiarities of these would with no shadow of a probability be repeated in any other person. Doubtless in minute detail this would be correct; but whether the distinctive features would be clearly defined by a bite upon the skin, a very movable and flexible substance with soft cellular tissue, together with soft, irregular muscles beneath it, and these, too, lying over and around irregularly shaped bones as the ulna and radius are at this point, is very questionable, indeed. Had the indentations, that must have been made at the time of the injury, remained till the examination, well defined, and the correspondence between these and the teeth of the prisoner or a well prepared cast of them been complete, it would have been a much stronger case, indeed, would have amounted to almost a certainty, as to the fact; but the time when, would have been quite another matter.

There was much questioning and quibbling in regard to the possibility of conveying the distinctive features of the teeth by impression in softened beeswax. Every one of much experience will at once recognize the fact that some of the peculiarities will be thus unmistakably reproduced; of course, many errors may be made by faulty manipulation.

In all such matters as the above it will be readily conceded that there is a great diversity of perception. This diversity is innate, and is many a time modified by prejudice, desire, or preconceived opinions.

The question as to whether there is an unmistakable identity in the teeth of each and every individual is one about which close observers would not differ, but whether that individuality would be clearly marked by the impressions made on soft substances by the edges and masticating surfaces of the teeth, is a question about which there is room for diversity of opinion. If, however, such impression was made by a full set of teeth, or any considerable number of them, it would be very rare, indeed, that there would be a perfect correspondence between that and any other set of teeth, in all the following particulars, viz: the size of the circle occupied by the teeth, its curvature, the relative position of the ends of the teeth in respect to elongation, and in respect to torsion, and inward and outward displacement, and in addition to all this the variations in form of the edges and ends of the teeth, the thick or thin, sharp or blunt, broad or narrow, large, sharp, well defined prominences or cusps, with deep, irregular indentations and grooves about them, or smooth, level surfaces, with sharp or rounded corners; in general shape well defined and uniform or irregular.

Now in all these respects no good observer will ever expect to find perfect or any thing very near a perfect similarity between any two cases.

We have referred to this case to show how much importance attaches or may attach in a legal aspect to the individuality of the teeth, and the part they may play in the phases of life.

